# Love Wave Surface Acoustic Wave Sensor with Laser-Deposited Nanoporous Gold Sensitive Layer

**DOI:** 10.3390/s19204492

**Published:** 2019-10-16

**Authors:** Cristian Viespe, Valentina Dinca, Gianina Popescu-Pelin, Dana Miu

**Affiliations:** National Institute for Laser, Plasma and Radiation Physics, Laser Department, Atomistilor #409, 077125 Bucharest-Magurele, Romania; cristian.viespe@inflpr.ro (C.V.); valentina.dinca@inflpr.ro (V.D.); gianina.popescu@inflpr.ro (G.P.-P.)

**Keywords:** SAW sensor, pulsed laser deposition, gas sensor, Au, nanoporous film, Love Wave

## Abstract

Laser-deposited gold immobilization layers with different porosities were incorporated into Love Wave Surface Acoustic Wave sensors (LW-SAWs). Acetylcholinesterase (AChE) enzyme was immobilized onto three gold interfaces with different morphologies, and the sensor response to chloroform was measured. The response of the sensors to various chloroform concentrations indicates that their sensing properties (sensitivity, limit of detection) are considerably improved when the gold layers are porous, in comparison to a conventional dense gold layer. The results obtained can be used to improve properties of SAW-based biosensors by controlling the nanostructure of the gold immobilization layer, in combination with other enzymes and proteins, since the design of the present sensor is the same as that for a Love Wave biosensor.

## 1. Introduction

Gas sensors are used in numerous domains such as the detection of Chemical Warfare Agents (CWA), control of emissions or monitoring of various hazardous gases [1,2]. For example, chlorinated hydrocarbons, (i.e., chloroform) are known for their toxic effect and damages to liver and kidneys, e.g., acute and chronic intoxication from exposure to chloroform in industry [3,4]. Several types of gas sensors are currently used, such as resistive sensors, optical devices or Surface Acoustic Wave sensors (SAWs) [5,6,7]. Among them, SAW sensors present a series of advantages such as high sensitivity, fast response, reliability, ease of fabrication, and low cost [7,8,9]. The sensitivity is a result of the detection mechanism, which is based on perturbation of the SAW propagation, primarily by mechanical or acoustoelectric effects, in the presence of the analyte [10].

In addition to applications in gas sensing, SAW sensors have also been used in Love Wave (LW) type biosensors, which have the considerable advantage of allowing label-free recognition when combined with biological receptors [11,12,13]. In this case, the SAW sensor structure is modified to adapt it to operation in a liquid environment, to avoid strong damping of the surface wave in the presence of liquid media [11,14]. A guiding layer is deposited on top of the piezoelectric substrate, which traps the acoustic energy near the sensing surface, ensuring operation in contact with liquid media and increasing sensitivity [15]. A LW is a horizontally polarized shear wave which is guided in a layer with a shear velocity lower than the piezoelectric substrate, and propagates in the guiding layer and in the part of the substrate close to the interface. The physical principle of the detection is in this case the variation of the mass and visco-elastic properties of the receptor material of the sensor as a result of bonding of biological material (biomolecules, proteins, peptides, etc.) on its surface [16]. The sensing properties of a biosensor are related both to the efficiency of the bonding of the biological material to the receptor material, and to the response of the sensor to the variation of the properties of the receptor material which results from this bonding. Gold is widely used in SAW biosensors as the immobilization layer, since it has properties making it suitable for the detection of a large range of analytes [17,18].

Nanoporous gold is of great interest for sensors due to properties such as high surface to volume ratio, chemical, and physical stability and biocompatibility. It has been used both alone and in combination with an additional layer such as an enzyme on its surface to detect gases or biological samples. For example, electrochemical sensors with nanoporous gold in combination with enzymes were used to detect H_2_O_2_, or lipids such as cholesterol and triglycerides [19,20]. Nanoporous gold was also used in a Quartz Crystal Microbalance, where an increase of the sensitivity by a factor of 40 was reported by using porous gold instead of dense, thermally evaporated gold [21,22]. However, to our best knowledge, no use of nanoporous gold in SAW sensors has been reported. Gold has been used in SAW sensors both for gas sensing [23,24] and in biosensors [18,25], but only as a dense film. On the other hand, research has demonstrated considerable improvement of the gas sensing properties of SAW sensors when various nanoporous sensing layers such as oxides, metals or carbon nanotubes are used [7,26,27]. An increase of the specific surface of gold used in SAW gas sensors or in biosensors can therefore be expected to improve their sensitivity, as well.

Pulsed Laser Deposition (PLD) is a method of obtaining thin films that has the advantage of permitting relatively facile control of the film morphology [28]. The morphology of films deposited by PLD can easily be controlled by variation of primary deposition parameters such as deposition pressure and geometry, laser pulse energy, or number of laser pulses [29,30]. In particular, engineering of the nanostructure of gold films is possible by using PLD [28,31,32].

Acetylcholinesterase (AChE) in an enzyme known for its significant role in signal termination in the cholinergic system, which has recently been used as an active element for a variety of biosensors and chemical sensors. For example, the AChE enzyme immobilized by physical adsorption onto different surfaces (i.e., silica sol-gel incorporating gold nanoparticles (AuNPs-Si-SG), platinum-coated with AChE) was used for clinical applications of therapeutic drugs (donepezil, rivastigmine, huperzine, galantamine) or in screening and testing for neurological diseases (e.g., Parkinson’s and Alzheimer’s) [33,34,35,36,37]. Other applications focused on the detection of pesticides (carbofuran, malaoxon, malathion) or CWA (sarin, soman, tabun, VX); new approaches implied embedding the enzymes within a polymeric layer for detection of dimethyl methylphosphonate (DMMP) and diisopropyl methylphosphonate (DIMP) [38].

The present paper reports the sensing properties of SAWs based on laser-deposited gold sensing films with various morphologies. As a proof of concept, AChE enzyme was immobilized onto three gold interfaces with different morphologies, and the sensor response to chloroform was measured. We have obtained considerable improvement of the sensing properties (sensitivity, limit of detection—LOD) of sensors with nanoporous gold compared to those based on dense gold thin films. The results obtained could be extended to other enzymes or proteins and used to improve the sensitivity of SAW-based biosensors. The sensor described in the present paper, based on nanostructured gold layer deposited on top of the Poly(methyl methacrylate) (PMMA) layer, has the potential to provide improved biosensing properties in comparison to the similar one with a dense gold layer.

## 2. Materials and Methods

The LW-SAW sensor is based on a 0.5 mm thick piezoelectric quartz crystal (Roditi International Corporation Ltd.; London, UK), Y-cut (42.75°), with propagation direction 90° with respect to the *x*-axis, cut in a parallelogram geometry to reduce unwanted SAW reflections. It is a delay-line type with an oscillating frequency of ≈69 MHz [16]. The SAW transducers were fabricated using photolithography and have Interdigital Transducers (IDTs) with Cr and Au metallization film thicknesses of 10 and 150 nm, respectively. Each input and output interdigital transducer consists of 50 electrode pairs, with an aperture width 2500 µm, and a wavelength of ~45 µm; the distance between IDTs was 10 mm.

To eliminate the influence of temperature on the SAW sensor oscillation frequency, a Peltier element was used to control the temperature to an accuracy of 0.01 K. The control system consisted of a 70 W Peltier element, a TEC-1089-SV bidirectional thermo-electric controller, a Pt100 temperature sensor, computer, software interface, and voltage source. Thus, all measurements of the sensors were made at constant temperature.

A layer of polymethylmethacrylate (PMMA) (Micro Resist Technology GmbH) was applied over the clean surface by spin coating. The PMMA solution was deposited according the following program: 100 rps—5 s, 500 rps—5 s, 1500 rps—20 s, 2500 rps—40 s, 1500 rps—20 s, 500 rps—20 s, in order to ensure a uniform deposition over the entire sensor surface. The solidification of the polymer occurred immediately after deposition, by gradual heating of the sensor up to 190 °C (in one hour). The thickness of the resulting PMMA layer is 2 µm. PMMA is frequently used in Love Wave-type SAW biosensors in order to avoid strong damping of the surface wave in a liquid environment due to its low shear wave acoustic velocity (1105 m/s), relatively low density (1.17 g/cm^3^), high stiffness module (1.7 GPa) and good elastic properties [10,16,39]. In addition, the PMMA has the role of protecting the interdigital electrodes, as well as of circumventing the well-known problem of poor adhesion of gold onto quartz. Although we only tested the SAW sensor design described here for gas (chloroform) sensing, it can also be used in a biosensor, due to properties such as biocompatibility, low moisture uptake, and minimal swelling in solution. Therefore, the design used in this case for chloroform detection is applicable for biosensors, as well.

Layers of Au were deposited by PLD in various conditions. A Nd-YAG laser (EKSPLA model NL301HT) with 5 ns pulse duration, at an emission wavelength of 532 nm was used, operating at 10 Hz repetition rate. Depositions were made in a vacuum chamber equipped with a gas pressure and flow control system, consisting of a mass flow controller on the gas bottle (mks 1179B Series) connected to a 4 Channel Programmer/Display (mks model 647C Multi Gas Controller), and an exhaust throttle valve (mks 253B-1-40-1) mounted on a rotary vane pump, and connected to a pressure controller (mks 651C-D2S1N). Depositions were made at various pressures between vacuum (10^−5^ Torr) and 4 Torr Ar gas.

The gold films were deposited onto substrates placed 4 cm from the target. All depositions were made at room temperature. The target was placed on computer-controlled x-y tables which ensure target movement to avoid erosion. Gold films were deposited onto silicon substrates for analysis of morphological properties, and onto the SAW sensors with the PMMA layer described above for measurement of the sensor properties. The gold films were deposited with various numbers of pulses, from 1000 to 50,000. The combination of variation of pressure and number of pulses led to variation of the morphology and porosity of the gold films.

The chemicals used for enzyme immobilization were obtained from Sigma-Aldrich (Saint Louis, MO, USA). A solution of 0.1 M phosphate buffer pH 8, containing 0.1% weight AChE (C3389 Type VI-S, lyophilized powder, 200–1000 units/mg protein) was homogenized and 500 µL solution was placed onto on the gold layer on the sensor surface, put in a humidity chamber and kept overnight at 4 °C. The AChE-modified surface was then washed 4× with phosphate buffer and 2× with deionized water, dried using nitrogen and kept at 4 °C prior to use.

The surface of the nanostructured gold films were investigated by Scanning Electron Microscopy (SEM) (Thermoscientific Apreo S, Waltham, MA, USA) and Atomic Force Microscopy (AFM) (Park System XE-100, Suwon, Korea). The roughness of the films was assessed using AFM. The surface of gold layers deposited onto Si in the same conditions as those deposited onto the sensors was also visualized by AFM after enzyme immobilization.

A frequency counter (Pendulum CNT-91) connected to a computer system with a Time View III (Pendulum Instruments, Banino, Poland) software, monitored the frequency change. Three sensors with different morphologies of the Au layers were tested towards chloroform at room temperature. Different concentrations of chloroform were injected into the gas mixer chamber. Using a diaphragm pump (Pfeiffer MVP 035-2), the mixture of chloroform and air, at a given concentration, was circulated in the testing system at a constant flow rate of 150 cm^3^/s. Sensor S1 had a dense Au layer which was deposited in vacuum. Sensors S2 and S3 were both deposited at a relatively high Ar pressure of 4 Torr and both had nanoporous Au layers; the different degree of porosity (roughness), of the two is due to the difference in deposition pulses, 9800 and 30,000, respectively. All three were tested for various chloroform concentrations, between 125 and 2000 ppm.

## 3. Results

### 3.1. Morphology

**Figure 1:**
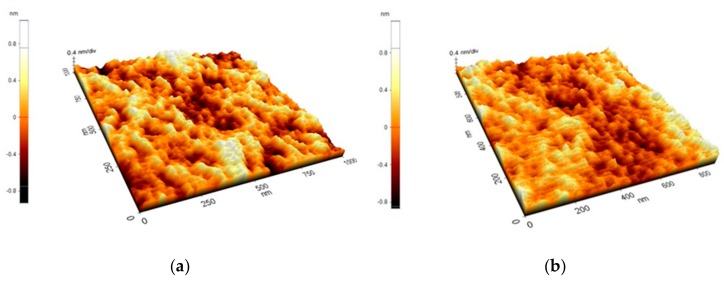
AFM images of the surface of a PMMA layer deposited on a quartz substrate (**a**), and that of a gold layer deposited on top of a PMMA/quartz layer (**b**). The gold layer was deposited in 450 mTorr Ar using 9800 laser pulses.

It has been reported that in certain conditions the morphology of polymer films can change through viscous flow, as a result of gold nanoparticles being sputter-deposited onto their surface [40,41]. AFM analysis of the PMMA layers on quartz before and after deposition of the gold films have indicated that this is not the case in our conditions. As can be seen in Figure 1, the morphology of the surface was similar for the polymer surface before and after a gold layer was deposited on it. This result is for a relatively low deposition pressure (450 mTorr Ar), which implies a large energy of the gold species incident onto the polymer surface [28,32,42]. The nanoporous gold layers on the SAW sensors which were tested were deposited at a higher deposition pressure of 4 Torr, which leads to a lower energy of the incident species due to hydrodynamic effects in the laser ablation plasma [32]. Therefore, we consider that the morphology of the PMMA layer is not affected by the gold species deposited on top of it in the case of the sensors.

SEM images of the deposited films revealed that the morphology of the gold layer depends on the deposition pressure and the number of pulses, as has been reported by other researchers [28]. Larger pressures lead to hydrodynamic effects such as slowing of the target species, spreading of the ablation plasma plume over the substrate surface, as well as gas-phase nucleation of nanoparticles in the target-substrate region [43,44,45,46]. The gold species incident on the substrate have smaller energies as the pressure increases, generating less dense films [30,32]. For a relatively small number of laser ablation pulses, the gold is deposited in the form of separate nanoparticles; as the ablation continues, the nanoparticles form aggregates and, for lower pressures, continuous films. At increased pressures, even for many pulses no continuous films are formed [28].

**Figure 2:**
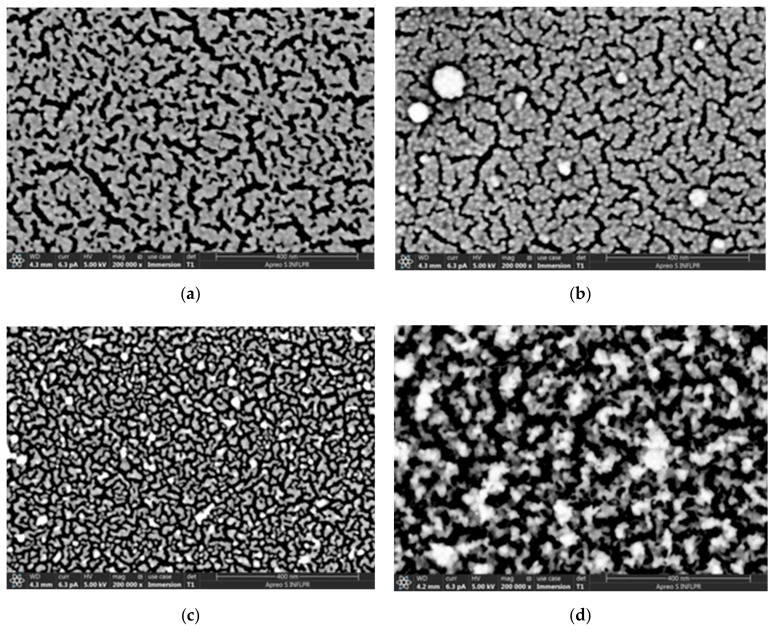
SEM images of the gold layers deposited directly onto Si substrates for high Ar deposition pressures and various numbers of ablation pulses. (**a**) 1 Torr, 9800 pulses; (**b**) 1 Torr, 30,000 pulses; (**c**) 4 Torr, 9800 pulses (same as Au layer in S2); (**d**) 4 Torr, 30,000 pulses (same as Au layer in S3). Scale bars are 400 nm in all cases.

**Figure 3:**
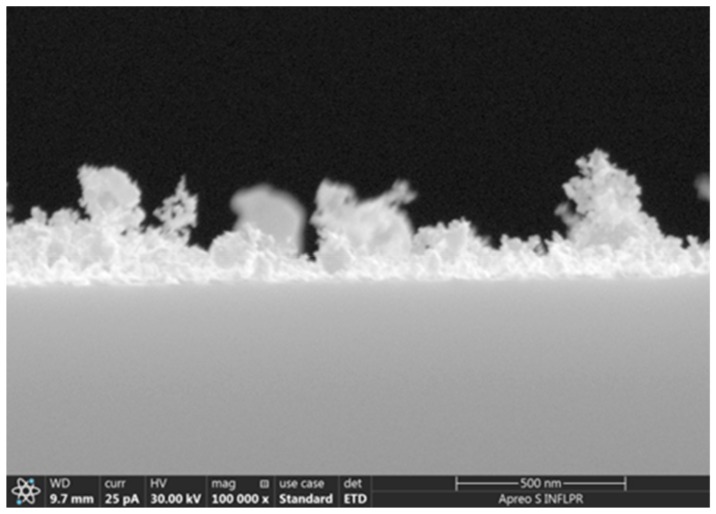
SEM image of the cross-section of a gold film deposited on Si at 4 Torr Ar pressure, using 30,000 laser ablation pulses. Corresponds to the film with the surface shown in Figure 2d. Scale bar is 500 nm.

In our case, at a high pressure (4 Torr) there were still separate nanoparticles after ablation by 1000 pulses. As the number of pulses increased, the Au species formed various agglomerations separated by cracks with widths from several nm to about 25 nm. The dimensions of these cracks were in the range of pore sizes reported for nanoporous gold successfully used in other types of biosensors [19]. The morphology of the films deposited at 4 Torr Ar differed considerably from the other pressures we used, both for 9800 and 30,000 pulses. At the highest pressure and number of ablation pulses used (4 Torr, 30,000 pulses), the Au layer deposited onto Si had a very porous appearance (Figure 2d). This is also evident in the SEM image observed in cross-section (Figure 3). The thickness of the very porous gold layer obtained in these conditions is hard to define; the layer consists of a denser layer about 50 nm thick, on top of which irregular, extremely porous structures of greatly variable thicknesses (70–400 nm) are visible. The morphology of the film changed considerably at 4 Torr as the number of ablation pulses increased and the gold layer became thicker. The gold layer which consisted of relatively compact agglomerations separated by cracks about 6–12 nm wide after 9800 ablation pulses became an extremely porous film with the width of pores between agglomerations of up to 25 nm after 30,000 pulses (Figure 2). Since the morphologies of these two layers deposited at 4 Torr differed considerably, they were chosen for comparison of the sensing properties. The properties of sensors with Au layers deposited onto PMMA/quartz, in the same conditions as the gold layers in Figure 2c,d (S2–Au layer deposited in 4 Torr, 9800 pulses; S3–Au layer deposited in 4 Torr, 30,000 pulses), in the presence of various chloroform concentrations were determined. These were compared to a sensor with a “classical” dense Au layer as reported for SAW sensors (S1, deposited with 2000 pulses). As determined by AFM, the roughnesses of the gold layers deposited in the same conditions as the three sensor samples were: 0.7 nm for the layer deposited in vacuum (same as S1), 6 nm for the layer deposited with 9800 pulses in 4 Torr Ar (as S2), and 30 nm for 4 Torr 30,000 pulses (as S3). The thickness of the dense Au layer in S1 is about 40 nm, and that in sensor S2 is about 15 nm. As already mentioned, the thickness of the very porous Au layer in S3 is hard to define due to its irregular surface.

### 3.2. Sensor Properties

The immobilization of the enzyme onto the gold layer is characterized by the frequency shift of the sensors. The response of the sensor after AChE immobilization on the three sensor samples revealed that a larger quantity of enzyme is loaded onto the porous surfaces (S2 and S3) than onto the dense one (S1). Sensor S1, which had a dense and relatively smooth Au layer, had a frequency shift of 9 kHz as a result of enzyme immobilization. Sensor S2 had a morphology characterized by agglomerations of nanoparticles separated by cracks, and a frequency shift of 24 kHz after enzyme immobilization. Sensor S3, obviously the most porous one, had a frequency shift of 26 kHz in the same conditions as the other two sensors. There is a large difference in frequency shift between the sensor with the dense layer (S1) and the porous ones (S2 and S3). However, the difference between the two porous sensors is less than 10% (frequency shift, therefore enzyme binding), even though the difference in roughness of the gold layer is considerable between S2 and S3 (6 nm vs. 30 nm, respectively). It is worth mentioning, however, that although the difference in the frequency shifts caused by enzyme immobilization onto S2 and S3 is small, it is larger than the measurement error.

**Figure 4:**
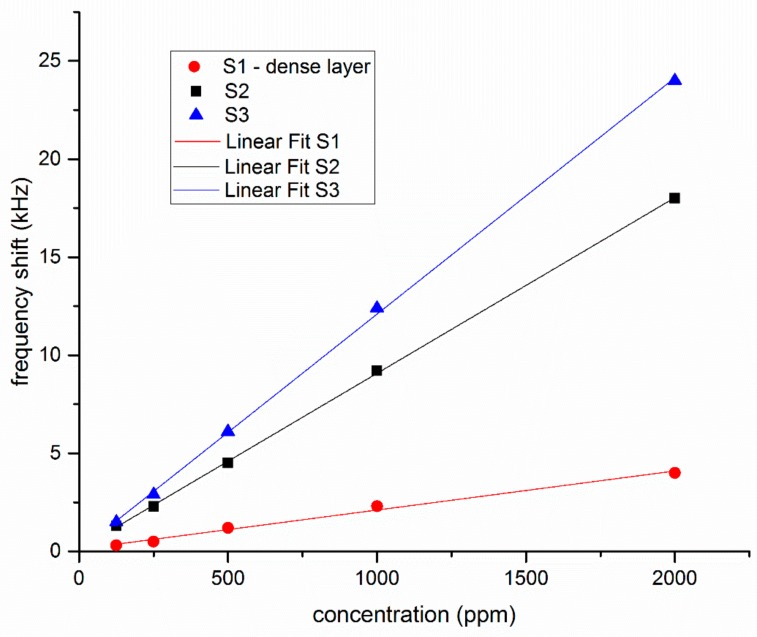
Frequency shifts of sensors S1, S2, and S3, with gold layers deposited in different conditions as described in text, for various concentrations of chloroform.

The response of the three sensors towards various concentrations of chloroform (125 and 2000 ppm) are presented in Figure 4 (the data points correspond to one measurement at the given concentration). As the figure shows, the two sensors with nanoporous Au layers have much higher frequency shifts than the sensor with the dense Au layer. The response of all three sensors is linear in the concentration domain that was studied, without any indication of saturation. The fact that the response of S2 and S3 is higher than that of S1 is to be expected, since the frequency shifts upon enzyme immobilization indicates that a larger mass of enzyme was immobilized onto nanoporous gold layers than onto the dense one.

The values of the frequency shifts, for chloroform concentrations between 125 and 2000 ppm were used to determine the average value of the sensitivity and LOD of the sensors, given in Table 1. The sensitivity represents the frequency shift in Hz per unit analyte concentration in ppm. The LOD is defined as three times the noise level per sensitivity [10].

## 4. Discussion and Conclusions

**Figure 5:**
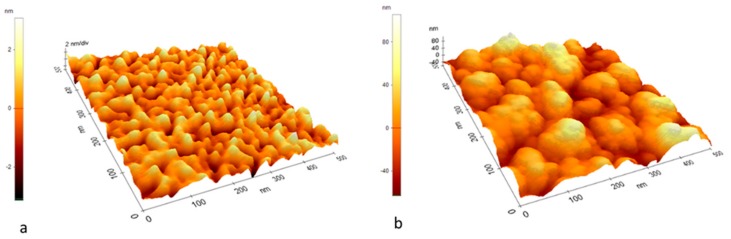
AFM image of the AChE enzyme on the S2 (**a**) and S3 (**b**) sensor surface. The roughness per area measured 500 nm × 500 nm is 1.1 nm and 37 nm, respectively.

**Figure 6:**
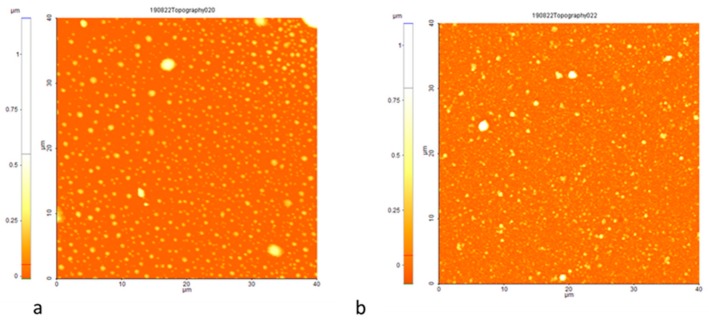
AFM image of the AChE enzyme on the S2 (**a**) and S3 (**b**) sensor surface over large surfaces (40 µm × 40 µm).

Table 1 reveals that the properties of the sensors with nanoporous gold layers are much better than the one with a dense layer. However, the difference between the two porous layers is smaller than one would expect from their morphology and the value of their roughness. This may be because it is not only the amount of enzyme which is immobilized onto the surface which affects the sensing properties, but also its morphology and roughness. It is known that the AChE binding on the gold layer can occur through the nonfunctional region of the enzyme, and therefore the interference with or blocking of the active site of the enzyme is minimum. The esteratic subsite (Ser-His-Glu) and the peripheral binding anionic subsite of AChE gives the ability to bind to many different types of ligands, including chlorinated compounds [47]. Therefore, when exposing the sensor to chloroform vapors, the responses must be correlated with the sensor interface characteristics after immobilization, as well. For a better visualization of the modifications induced by enzyme binding, the topography and roughness analysis of the sensor surface were analyzed on both small scale (500 nm × 500 nm) (Figure 5) and larger scale (40 µm × 40 µm) (Figure 6) after enzyme immobilization. The AFM analysis of the surfaces with the enzyme reveal significant differences in roughness for S2 and S3: 1.1 nm for S2, respectively 37 nm for S3 (Figure 5). The presence of nanocracks in the case of S2 could lead to an accumulation of the enzyme within them, blocking the interior of the interface porosity, and decreasing the roughness in comparison to the initial S2 gold surface. In the case of S3 types of surfaces, characterized by larger structures, a binding of the enzyme could occur only onto the surface, without blocking it within pores/cracks.

On the other hand, the roughness area mediated over large surfaces (40 µm × 40 µm) for S2 and S3 after enzyme immobilization is influenced by the presence of randomly distributed island-like material, so that the differences in values are smaller for the two types of surfaces (56 nm for S2, respectively 75 nm for S3). It is observed in Figure 6 that the percentage of islands with tens of nm is higher on the surface of S2 (Figure 6a), while in the case of S3 (Figure 6b), the islands are distributed more randomly, with predominant rough structures as observed previously. This could offer some indication for the fact that the sensing properties of sensors S2 and S3 are not as different as the nanostructure of the gold layers they incorporate could suggest.

In conclusion, Love Wave-type SAW sensors based on AChE enzyme/nanoporous gold/PMMA/quartz were tested towards chloroform detection. The morphology of the gold layers was controlled through the PLD deposition conditions, namely the deposition pressure and the number of ablation pulses. Sensors with nanoporous gold layers had a considerably better response to chloroform concentrations between 125 and 2000 ppm, showing improved sensitivity and LOD in comparison to a conventional sensor based on a dense gold layer.

Although the difference between the roughness of the gold films for the two sensors with nanoporous layers was large, and was reflected in the mass of enzyme immobilized onto the gold surface, the difference in sensing properties was smaller than would be expected. This is probably because it is not only the amount of enzyme which is immobilized onto the surface which affects the sensing properties, but also its morphology and roughness.

To our best knowledge, it is for the first time that LW SAW sensors based on nanoporous gold were reported. The results we obtained can be used to improve properties of SAW-based biosensors by controlling the nanostructure of the gold immobilization layer, in combination with other enzymes or proteins, since the design of the present sensor is the same as that for a LW biosensor.

## Figures and Tables

**Table 1:** Sensitivity and LOD (Δf–frequency change, c-chloroform concentration) for the sensitive films.

LW-SAW Sensors	Sensitivity Δf/c Hz/ppm	LOD ppm
S1	2	41
S2	9	10
S3	12	7
